# *LRRK2* Loss‐of‐Function Variants in Patients with Rare Diseases: No Evidence for a Phenotypic Impact

**DOI:** 10.1002/mds.28452

**Published:** 2021-01-12

**Authors:** Christian Beetz, Ana Westenberger, Ruslan Al‐Ali, Najim Ameziane, Nadia Alhashmi, Rose‐Mary Boustany, Fuad Al Mutairi, Majid Alfadhel, Zuhair Al‐Hassnan, Moenaldeen AlSayed, Krishna K. Kandaswamy, Omid Paknia, Volha Skrahina, Arndt Rolfs, Peter Bauer

**Affiliations:** ^1^ CENTOGENE GmbH Rostock Germany; ^2^ Institute of Neurogenetics, University of Lübeck Lübeck Germany; ^3^ National Genetic Center Royal Hospital Muscat Oman; ^4^ Neurogenetics Program, AUBMC Special Kids Clinic and Division of Pediatric Neurology, Department of Pediatrics and Adolescent Medicine American University of Beirut Medical Center Beirut Lebanon; ^5^ Department of Biochemistry and Molecular Genetics American University of Beirut Medical Center Beirut Lebanon; ^6^ Genetics and Precision Medicine Department King Abdullah Specialist Children Hospital, King Abdulaziz Medical City, MNGHA Riyadh Saudi Arabia; ^7^ King Abdullah International Medical Research Center (KAIMRC) King Saud bin Abdulaziz University for Health Sciences, MNGHA Riyadh Saudi Arabia; ^8^ Department of Medical Genetics King Faisal Specialist Hospital and Research Centre Riyadh Saudi Arabia; ^9^ College of Medicine Alfaisal University Riyadh Saudi Arabia; ^10^ University of Rostock Medical Faculty Rostock Germany

Certain missense variants in the *LRRK2* gene, which encodes leucine‐rich repeat kinase 2 (LRRK2), are a common cause of Parkinson's disease (PD).^1^ As these variants increase the protein's kinase activity, treatment strategies aim at reducing LRRK2 function and/or abundance. Safety concerns for this approach are based on considerable *LRRK2* expression in numerous tissues and organs.^2^ Indeed, LRRK2 knockout/inhibition in mammalian model organisms has been reported to affect the lungs, the kidneys, and the liver.^3^ Findings from two recent studies on *LRRK2* loss‐of‐function (LoF) variants in humans have been interpreted as partially alleviating pertinent concerns: Blauwendraat and colleagues showed absence of enrichment of these variants in PD patients,^4^ while Whiffin and colleagues revealed that their presence is not associated with reduced life expectancy, abnormal standard laboratory parameters, or common adverse phenotypes in a large cohort of healthy individuals.^5^ However, the probably most relevant human subjects, that is, those presenting with (rare) disease phenotypes, have not yet been systematically analyzed. The present study describes the spectrum and frequency of *LRRK2* LoF variants in CentoMD®, a data repository for patients with rare disorders,^6^ and analyzes the dataset for potential disease associations.

*LRRK2* sequencing data from over 70,000 individuals (45,331 patients; 25,043 healthy family members) from diagnostic exome or genome sequencing were interrogated. In 154 cases, good quality^7^ *LRRK2* LoF variants were present. There were a total of 52 distinct variants (22 nonsense, 20 frameshift, 10 splice‐site) (Fig. S1). The fraction of patients was 0.636 among variant‐positives and 0.644 among variant‐negatives, demonstrating absence of an association of *LRRK2* LoF variants with disease. Focusing on only patients, there was no significant difference between variant‐positives and variant‐negatives with respect to age at referral and number of Human Phenotype Ontology (HPO) terms per patient (Fig. 1A,B). Furthermore, none of the HPO terms that were most frequently observed in *LRRK2* LoF‐positives was significantly enriched in this cohort (Fig. 1C). Thus, rare disease patients with *LRRK2* LoF variants are not affected earlier or more severely than other patients, and do not have specific phenotypes.

**FIG. 1:**
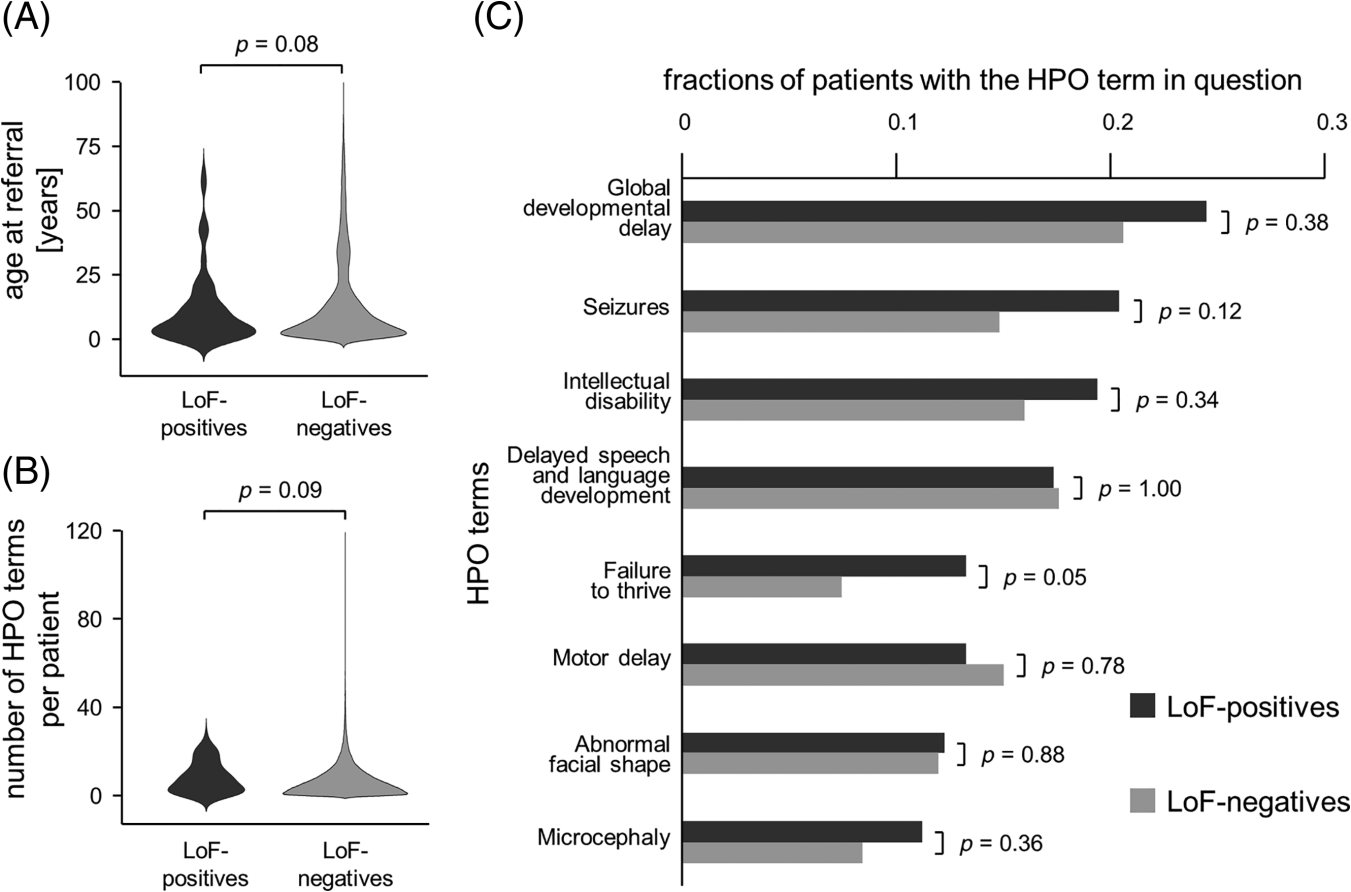
Comparison of *LRRK2* LoF‐positive and *LRRK2* LoF‐negative patients. (**A**) Age at referral. (**B**) Number of Human Phenotype Ontology (HPO) terms per patient. (**C**) Fractions of patients with HPO terms that are present in at least 10% of *LRRK2* LoF‐positive patients. *P* values are two‐tailed and are not corrected for multiple testing; they are based on Student's t‐test in (**A**) and (**B**), and on Fisher's exact test in (**C**).

Of note, four of the *LRKK2* LoF variant‐positives were homozygous (Table S1; Fig. S2), of whom three were patients who did not show any obvious phenotypic overlap. The first patient had received a negative genetic report, while a homozygous variant of uncertain significance was reported in the second and a homozygous pathogenic variant in the third. Importantly, the fourth *LRRK2* LoF homozygous individual was a 32‐year‐old healthy father of a patient. Even complete absence of LRRK2 may thus have no overt early phenotypic impact in humans, but further studies are necessary to determine whether the above individuals are indeed “human *LRRK2* knockouts.”

Our observations in a large and clinically diverse diagnostic cohort represent conceptually novel support for the safety of currently pursued therapeutic strategies for patients with kinase‐activating *LRRK2* mutations. Considering the accumulating evidence for an implication of increased LRRK2 activity also in the pathogenesis of other monogenic and idiopathic forms of PD,^1^ our findings are of potentially even broader relevance.

## Author Roles

(1) Research Project: A. Conception, B. Organization, C. Execution; (2) Statistical Analysis: A. Design, B. Execution, C. Review and Critique; (3) Manuscript Preparation: A. Writing of the First Draft, B. Review and Critique.

C.B.: 1A, 1B, 1C, 2A, 2B, 3A, 3B

A.W.: 1B, 2C, 3A, 3B

R.A.‐A.: 1B, 1C, 2B, 2C, 3B

N. Ameziane: 1B, 1C, 2B, 2C, 3B

N. Alhashmi: 1B, 1C, 3B

R.‐M.B.: 1B, 1C, 3B

F.A.M.: 1B, 1C, 3B

M.A.: 1B, 1C, 3B

Z.A.‐H.: 1B, 1C, 3B

M.A: 1B, 1C, 3B

K.K.K.: 1B, 1C, 2B, 2C, 3B

O.P.: 1B, 1C, 2C, 3C

V.S.: 1B, 1C, 2C, 3C

A.R.: 1A, 1B, 2A, 2C, 3B

P.B.: 1A, 1B, 2A, 2C, 3A, 3B

## Financial Disclosures of All Authors (for the Preceding 12 Months)

Christian Beetz.

**Table:** 

Stock Ownership in medically‐related fields	
Intellectual Property Rights	
Consultancies	
Expert Testimony	
Advisory Boards	
Employment	CENTOGENE GMBH
Partnerships	
Contracts	
Honoraria	
Royalties	
Grants	
Other	

Ana Westenberger.

**Table:** 

Stock Ownership in medically‐related fields	
Intellectual Property Rights	
Consultancies	for CENTOGENE GmbH
Expert Testimony	
Advisory Boards	
Employment	University of Lübeck
Partnerships	
Contracts	
Honoraria	
Royalties	
Grants	
Other	

Ruslan Al‐Ali.

**Table:** 

Stock Ownership in medically‐related fields	
Intellectual Property Rights	
Consultancies	
Expert Testimony	
Advisory Boards	
Employment	CENTOGENE GMBH
Partnerships	
Contracts	
Honoraria	
Royalties	
Grants	
Other	

Najim Ameziane.

**Table:** 

Stock Ownership in medically‐related fields	
Intellectual Property Rights	
Consultancies	
Expert Testimony	
Advisory Boards	
Employment	CENTOGENE GMBH
Partnerships	
Contracts	
Honoraria	
Royalties	
Grants	
Other	

Nadia Al‐Hashmi.

**Table:** 

Stock Ownership in medically‐related fields	
Intellectual Property Rights	
Consultancies	
Expert Testimony	
Advisory Boards	
Employment	Royal Hospital, Muscat, Oman
Partnerships	
Contracts	
Honoraria	
Royalties	
Grants	
Other	

Rose‐Mary Boustany.

**Table:** 

Stock Ownership in medically‐related fields	
Intellectual Property Rights	
Consultancies	
Expert Testimony	
Advisory Boards	
Employment	American University of Beirut Medical Center, Beirut, Lebanon
Partnerships	
Contracts	
Honoraria	
Royalties	
Grants	
Other	

Fuad Al‐Mutairi.

**Table:** 

Stock Ownership in medically‐related fields	
Intellectual Property Rights	
Consultancies	
Expert Testimony	
Advisory Boards	
Employment	King Abdulaziz Medical City, MNGHA, Riyadh, Saudi Arabia
Partnerships	
Contracts	
Honoraria	
Royalties	
Grants	
Other	

Majid Al‐Fadhel.

**Table:** 

Stock Ownership in medically‐related fields	
Intellectual Property Rights	
Consultancies	
Expert Testimony	
Advisory Boards	
Employment	King Abdulaziz Medical City, MNGHA, Riyadh, Saudi Arabia
Partnerships	
Contracts	
Honoraria	
Royalties	
Grants	
Other	

Zuhair Al‐Hassnan.

**Table:** 

Stock Ownership in medically‐related fields	
Intellectual Property Rights	
Consultancies	
Expert Testimony	
Advisory Boards	
Employment	King Faisal Specialist Hospital and Research Centre, Riyadh, Saudi Arabia
Partnerships	
Contracts	
Honoraria	
Royalties	
Grants	
Other	

Moeenaldin Al‐Sayed.

**Table:** 

Stock Ownership in medically‐related fields	
Intellectual Property Rights	
Consultancies	
Expert Testimony	
Advisory Boards	
Employment	King Faisal Specialist Hospital and Research Centre, Riyadh, Saudi Arabia
Partnerships	
Contracts	
Honoraria	
Royalties	
Grants	
Other	

Krishna K. Kandaswamy.

**Table:** 

Stock Ownership in medically‐related fields	
Intellectual Property Rights	
Consultancies	
Expert Testimony	
Advisory Boards	
Employment	CENTOGENE GMBH
Partnerships	
Contracts	
Honoraria	
Royalties	
Grants	
Other	

Omid Paknia.

**Table:** 

Stock Ownership in medically‐related fields	
Intellectual Property Rights	
Consultancies	
Expert Testimony	
Advisory Boards	
Employment	CENTOGENE GMBH
Partnerships	
Contracts	
Honoraria	
Royalties	
Grants	
Other	

Volha Skrahina.

**Table:** 

Stock Ownership in medically‐related fields	
Intellectual Property Rights	
Consultancies	
Expert Testimony	
Advisory Boards	
Employment	CENTOGENE GMBH
Partnerships	
Contracts	
Honoraria	
Royalties	
Grants	
Other	

Arndt Rolfs.

**Table:** 

Stock Ownership in medically‐related fields	Yes
Intellectual Property Rights	Yes
Consultancies	CENTOGENE GMBH
Expert Testimony	
Advisory Boards	
Employment	CENTOGENE GMBH
Partnerships	Yes
Contracts	
Honoraria	
Royalties	
Grants	Yes
Other	

Peter Bauer.

**Table:** 

Stock Ownership in medically‐related fields	Yes
Intellectual Property Rights	Yes
Consultancies	Yes
Expert Testimony	
Advisory Boards	
Employment	CENTOGENE GMBH
Partnerships	Yes
Contracts	
Honoraria	
Royalties	
Grants	Yes
Other	

## Supporting information


**Figure S1** *LRRK2* loss‐of‐function variants (description based on NM_198578.3) as identified in CentoMD®, and number of corresponding variant‐positive individuals

**Figure S2** Confirmation of homozygosity for three LRRK2 loss‐of‐function variants by Sanger sequencing (compare Suppl. Table 1). Affected nucleotides are marked by an arrow

**Table S1** Details for four individuals found to be homozygous for a *LRRK2* loss‐of‐function variant
