# Supplementary figures and images for: LRRK2 Loss‐of‐Function Variants in Patients with Rare Diseases: No Evidence for a Phenotypic Impact
Source: Mov Disord. 2021 Jan 12;36(4):1029–31. doi: 10.1002/mds.28452 (PMC8248088; doi:10.1002/mds.28452)

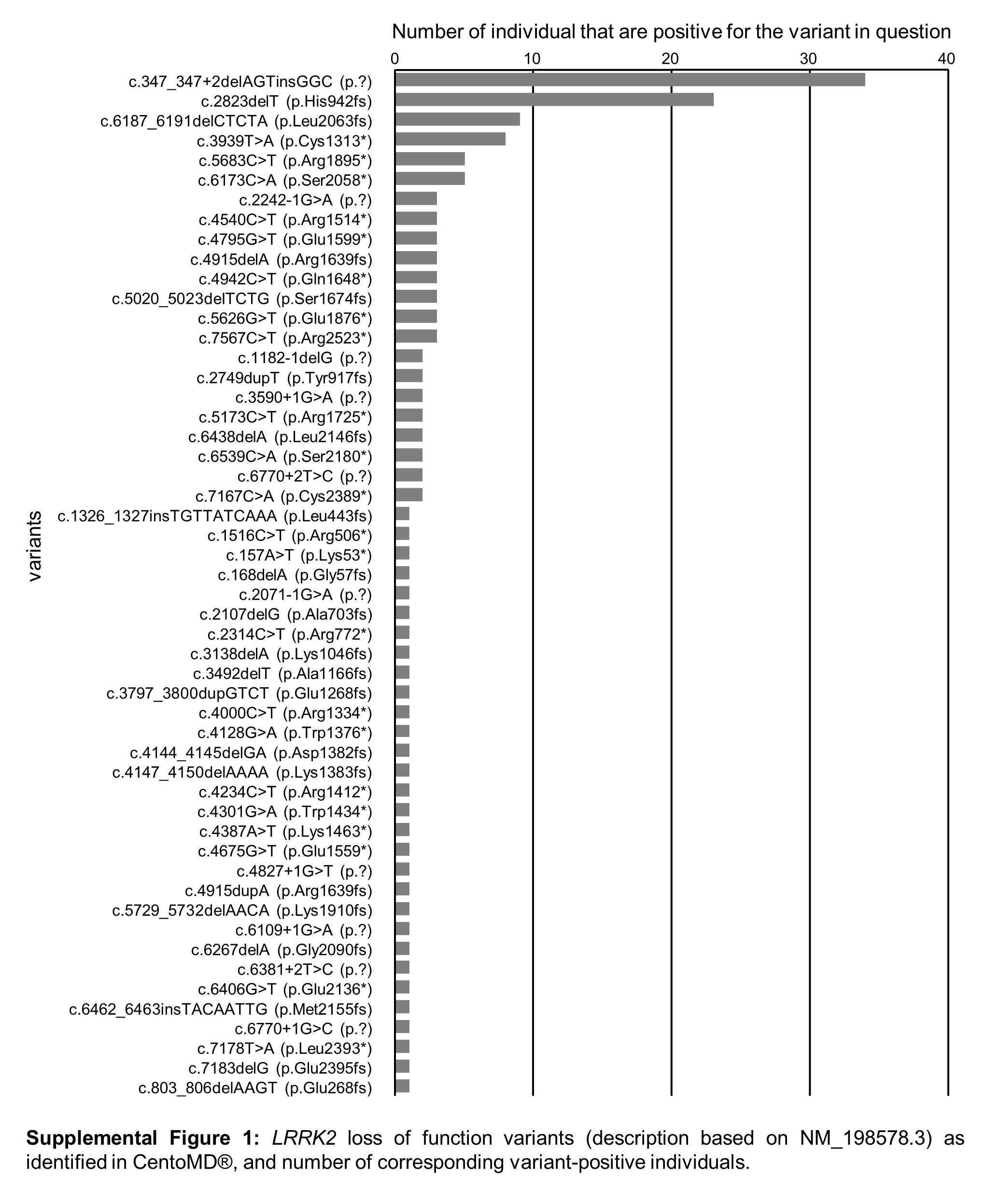

Supplement: Supplementary file 1 — Figure S1 LRRK2 loss‐of‐function variants (description based on NM_198578.3) as identified in CentoMD®, and number of corresponding variant‐positive individuals [file MDS-36-1029-s001.tif]

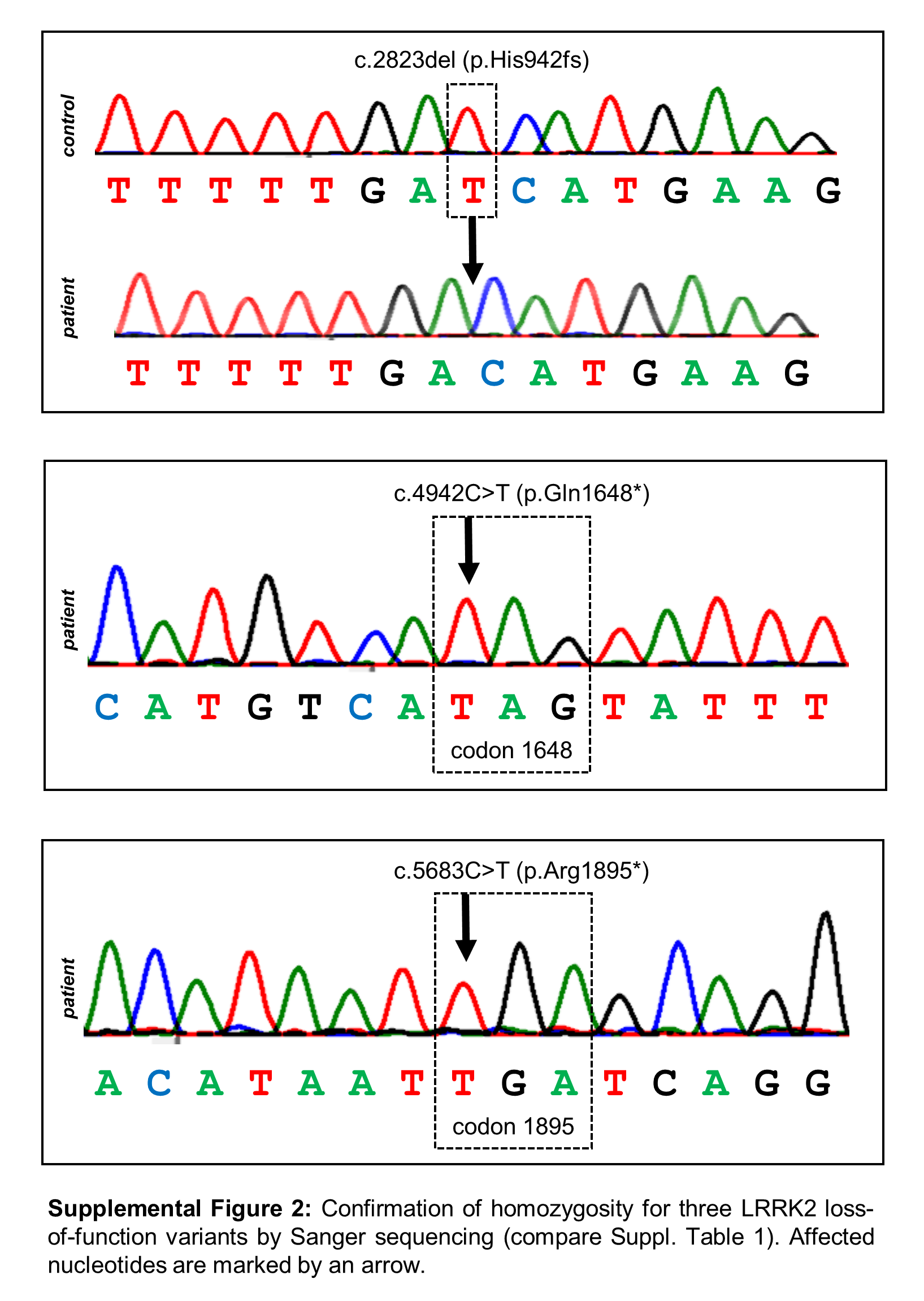

Supplement: Supplementary file 2 — Figure S2 Confirmation of homozygosity for three LRRK2 loss‐of‐function variants by Sanger sequencing (compare Suppl. Table 1). Affected nucleotides are marked by an arrow [file MDS-36-1029-s002.tif]
