# Supplementary material for: LRRK2 Loss‐of‐Function Variants in Patients with Rare Diseases: No Evidence for a Phenotypic Impact
Source: Mov Disord. 2021 Jan 12;36(4):1029–31. doi: 10.1002/mds.28452 (PMC8248088; doi:10.1002/mds.28452)
Supplement: Supplementary file 3 — Table S1 Details for four individuals found to be homozygous for a LRRK2 loss‐of‐function variant [file MDS-36-1029-s003.docx]

**Supplemental Table 1.** Details for four individuals found to be homozygous for a *LRRK2* loss-of-function variant.

| ***LRRK2* variant observed in homozygosity** | **gender** | **age**  **[years]** | **Human phenotype ontology terms** | **genetic diagnosis** |
| --- | --- | --- | --- | --- |
| c.2823del  (p.His942fs) | female | 19 | Abnormality of the coagulation cascade, Chronic hepatitis, Elevated hepatic transaminase, Failure to thrive, Hepatic encephalopathy, Hepatic failure, Hyperbilirubinemia, Hypoglycemia, Jaundice, Metabolic acidosis, Vomiting | Dihydrolipoamide dehydrogenase deficiency due to homozygousity for a pathogenic variant in *DLD* |
|  | male | 1 | Abnormal CNS myelination, Abnormality of eye movement, Abnormality of the cerebral white matter, Cognitive impairment, Developmental regression, Dysphagia, EEG abnormality, Failure to thrive, Feeding difficulties, Generalized myoclonic seizures, Global developmental delay, Growth delay, Hypertonia, Intellectual disability, Leukodystrophy, Limb dystonia, Motor delay, Neurodegeneration, Nystagmus, Reduced visual acuity, Rigidity, Seizures, Visual impairment | Possible autosomal recessive childhood-onset spasticity with hyperglycinemia due to homozygous variant of uncertain significance in *GLRX5* |
| c.4942C>T  (p.Gln1648*) | female | 5 | Abnormal circulating long-chain fatty-acid concentration, Abnormality of macular pigmentation, Abnormality of retinal pigmentation, Blindness, Bull's eye maculopathy, Global developmental delay, Mitochondrial myopathy, Muscular hypotonia, Seizures | none reached  (negative genetic report) |
| c.5683C>T  (p.Arg1895*) | male | 32 | none  (healthy father sent for diagnostic exome sequencing in a trio analytic setting) | N/A |
